# The role of social position and depressive symptoms in adolescence for life-course trajectories of education and work: a cohort study

**DOI:** 10.1186/s12889-016-3820-4

**Published:** 2016-11-18

**Authors:** Evelina Landstedt, Anna Brydsten, Anne Hammarström, Pekka Virtanen, Ylva B. Almquist

**Affiliations:** 1 Department of Public Health and Clincial Medicine, Epidemiology and Global Health, Umeå University, Umeå, Sweden; 2 Centre for Health Equity Studies (CHESS) Stockholm University/Karolinska Institutet, Stockholm, Sweden; 3 School of Health Sciences, University of Tampere, Tampere, Finland; 4 Department of Public Health and Caring Sciences, Uppsala University, Uppsala, Sweden

**Keywords:** Life course, Depressive symptoms, Sequence analysis, Social position, Social reproduction, Sweden, Trajectory

## Abstract

**Background:**

While a vast amount of studies confirm the social reproduction of class and status from one generation to the next, less is known about the role of health in the child generation for these processes. Research has shown that particularly mental distress in adolescence is important for future life chances. This study aimed to examine the importance of parental socioeconomic position and depressive symptoms in youth for life-course trajectories of education and labour market attachment among men and women.

**Methods:**

Based on four waves of questionnaire data from the Northern Swedish Cohort (*n* = 1,001), consisting of individuals born in 1965, three steps of gender-separate analyses were undertaken. First, the individual trajectories of education and labour market attachment from age 18 to 42 were mapped through sequence analysis. Second, cluster analysis was used to identify typical trajectories. Third, two indicators of parental socioeconomic position – occupational class and employment status – and depressive symptoms at age 16 were used in multinomial regression analyses to predict adult life-course trajectories.

**Results:**

Four typical trajectories were identified for men, of which three were characterised by stable employment and various lengths of education, and the fourth reflected a more unstable situation. Among women, five trajectories emerged, characterised by more instability compared to men. Low parental occupational class and unemployment were significantly associated with a higher risk of ending up in less advantaged trajectories for men while, for women, this was only the case for occupational class. Youth levels of depressive symptoms did not significantly differ across the trajectories.

**Conclusions:**

This study found support for the intergenerational reproduction of social position, particularly when measured in terms of parental occupational class. Youth depressive symptoms did not show clear differences across types of trajectories, subsequently impeding such symptoms to trigger any selection processes. While this could be a consequence of the specific framework of the current study, it may also suggest that depressive symptoms in youth are not a root cause for the more complex processes through which how social position develops across life. The possible impact of welfare and labour market policies is discussed.

## Background

The concept of social reproduction implies that the life courses of one generation are to some extent influenced by the life courses of previous generations. A common approach here is to take the child generation as the starting point and ask: “Where did these children come from?” and “Where did they end up?” [1]. Thus, focus is put on the correlation between the parents’ social position in society during upbringing and the social position of their children as they reach adulthood themselves. There is a vast amount of studies confirming this intergenerational social reproduction of class and status [2–7]. For example, the levels of parental education, income, and social class are associated with the children’s adult socioeconomic positions and career paths [6, 8–11]. Young people’s socioeconomic background have also shown to predict (un)successful transitions from school to work [12]. A strong correlation does not only suggest that children tend to become similar to their parents, but that the overall distribution of resources in society is reproduced. However, none of the studies referred to above have examined the role of mental health status in youth for the intergenerational reproduction of socioeconomic status.

Since previous research suggests that mental distress in adolescence is especially detrimental for future living conditions, it is arguably important to clarify mental health-related social selection [13–15]. In the current study we therefore focus on depressive symptoms. The lack of research into the role of health selection for social reproduction has been recognised by scholars arguing that more attention needs to be paid to “health as a potential mechanism through which intergenerational transmission of economic status takes place” [16]. While this is evident in the case for simple parent-child correlations of social class, education, and income [2, 3], less is known about the importance of health with regard to more complex life-course trajectories that reflect how the social position evolves across youth and mid-adulthood. In the current study we focus on depressive symptoms and on life-course trajectories examined through statuses related to education and labour market attachment (LMA). We define labour market attachment as the formal relationship with work in terms of proximity to the labour force, ranging from full-time employment to inactivity, either voluntary or involuntary [17].

The inquiry into life-course trajectories spans over several disciplines and various statistical techniques are used, for example latent class analysis and sequence analysis [18]. Sociological research into life-course trajectories has typically been dominated by the study of discrete transitions through the method of event history analysis [19]. In the more recent study of complex and holistic life-course patterns, sequence analysis has been employed [19, 20]. Sequence analysis is fully non-parametric and has the advantage of being able to handle multi-categorical statuses across time [18], and therefore it is the method of choice also in the present study.

In sum, the present study focuses on typical trajectories of education and labour market attachment across ages 18 to 42 as well as the extent to which parental socioeconomic position (occupational class and employment status) and the individuals’ own level of depressive symptoms can predict which typical trajectory the individuals’ lives take. The study is situated in a Scandinavian welfare state context and is based on data from the Northern Swedish Cohort which consists of a thousand men and women born in 1965. Since the age-specific transitions studied here are difficult to separate from period and cohort effects originating from the time and place in which a specific cohort is situated, we present more details on the context and the Northern Swedish Cohort further below.

### Trajectories of education and labour market attachment

Existing follow-up studies on education and LMA in the Western part of the world have mainly focused on either the youth-to-adulthood transition regarding education and establishment at the labour market [12, 21], or trajectories of LMA in adulthood [17]. For example, the youth-to-adulthood transition is a phase of life characterised by age-specific transitions periods to enter the adult stage of life, such as education, moving out from the family home, and establishing the first labour market contact [22, 23]. The role of individualisation and uncertainty in how young people navigate through this time in life has been central topic in youth research [24–26]. Other major transitions are those occurring during adulthood, primarily regarding LMA and retirement [17, 27], as well as family formation [28, 29].

Longitudinal patterns of education and work have typically been mapped as individuals’ transitions from one state to another, using event history analysis [19, 29]. A question here is to which extent such mapping considers the heterogeneity of life courses, such as co-occurring transitions or the varying duration of the different states [30]. Accounting for this complexity requires specific techniques, such as sequence analysis [8, 18, 19]. Sequence analysis is an exploratory method to identify and classify patterns of social processes and events across time [19, 31]. The timing and ordering of events or statuses is central. As Assave and colleagues [30] argue: “The benefit of sequence analysis is that it enables us to study a complex set of life-course trajectories as they actually take place, providing ideal-types of trajectories that can be interpreted and analysed in a meaningful way both in terms of theoretical perspectives and policy implications”. The present study uses sequence analysis to outline individual trajectories of education and LMA, followed by cluster analysis to identify a typology of typical trajectories, in a sample of Swedish men and women born in 1965. These techniques are further described in the method section.

### The role of health selection for trajectories of education and work

According to the literature on health selection in the labour market, individuals with initial poor health may be less likely to improve their position on the labour market and are therefore at risk of occupying jobs with low pay and poor working conditions which, in turn, are risk factors for future poor health [32, 33]. While the support for this hypothesis is relatively solid when considering employment status [5, 34–37], unemployment [38, 39] as well as insecure employment [40], studies of class mobility have identified less strong or no support for health selection, especially when considering youth mental health status [32, 41, 42].

Previous studies showing detrimental effects of poor mental health in youth and young adulthood for later employment, educational status, and financial security, have primarily examined associations between two time points [14, 43–46] or defined social mobility crudely in terms of being upward, stable, or downward [37, 47, 48]. Furthermore, empirical results in support of the mental health-related social mobility hypothesis have mainly been limited to the phase of adulthood [34, 40, 47, 49], or focused on childhood mental health in relation to adult health [9, 16, 50–52]. An exception is a Finnish study that identified a pathway from poor psychosomatic health in youth (age 16) to lower levels of education in adulthood (age 32), and that parental as well as own socioeconomic status acted as causes and consequences of such symptoms [53]. Moreover, according to a study from Norway on predictors of young people’s different pathways into adulthood, girls with a low level of global self-esteem were more likely to enter the ‘early mothers with partners’ pathway than the high education path [11]. No associations between youth self-esteem and characteristics of pathways to adulthood were identified for boys [11].

### Aim of the study

Based on the above outlined review we conclude that there is a lack of long-term longitudinal research exploring the complexity of life-course trajectories related to education and work and how youth circumstances are related to typical trajectories. There is a particular shortage of studies examining the role of mental health, such as depressive symptoms, in youth for the reproduction of social position across generations.

The aim of the present study was therefore threefold:

1. To identify typical trajectories of education and labour market attachment (from age 18 to 42) in Swedish men and women.
2. To investigate whether parental socioeconomic position – measured as parental occupational class and employment status – and/or adolescent depressive symptoms (age 16) predict which type of trajectory an individual’s life take.
3. To examine the extent to which any association between parental socioeconomic position and life-course trajectories can be explained by depressive symptoms in adolescence.

## Methods

### Context

This study is situated within a Nordic, social-democratic welfare context, historically characterised by universalism and solidarity [54]. In contrast to other European countries, both compulsory and higher education are free of tuitions, supported by child benefits (up to age 16), followed by study grants (up to age 20) and for higher education there is a student loan system via the Swedish state to citizens. Moreover, poverty is buffered for by an income protection system as well as by housing benefits and social benefits. The work-family conflict is buffered for by a well-developed parental leave system (approximately 1.5 years supported by the state), kindergarten (from 1 year), free school meals in kindergarten to end of secondary education and legal right to part-time employment for working parents with children under 8 years of age. It is a costly, publicly funded welfare system, with high demands on full employment for all citizens in working age, and the majority of the welfare benefits are based on labour market participation and income [54–57]. As a buffer during unemployment there are both benefits and an active labour market policy aiming at re-employment. From a gender perspective, the Swedish labour market has a distinct feature of high labour market participation among women, but also a vertical and horizontal gender segregated labour market, e.g. women and men tend to be found in different occupations and in different positions [58, 59]. Gender differences are also found in the level of education and income, where women tend to perform better in all levels of education but have lower income and positions across the labour market career [58].

### Population and data collection

The setting of this study was a middle-sized industrial residential town in Northern Sweden during the time period from 1981 until 2008. The town was representative for Sweden as a whole in relation to socio-demographics, health status, and health behaviour among young people [60]. The active labour market policy for young people in Sweden during the early 1980s meant that no one under the age of 21 should be unemployed.

The Northern Swedish Cohort consists of all individuals attending their final year of compulsory school in 1981 (age 16, born in 1965) in a medium-sized town in Northern Sweden (*n* = 1,083) [60]. Apart from the survey conducted in 1981 (T1), this study utilises data from the data collections in 1986 (T2, age 21), 1995 (T3, age 30), and 2007 (T4, age 42). There was a 1983 wave as well but it was omitted in the current study as it did not include any information relevant for the purpose of this study. By the last wave in 2007 the cohort comprised a total of 1,001 individuals, representing 94% of the cohort participants who were still alive.

### Variables

#### Education and labour market attachment (ages 18–42)

Information about education and LMA was collected retrospectively at T2, T3, and T4; together forming sequences consisting of 57 states (covering ages 18 to 42). At T2, the respondents were asked to account for their educational or work-related activities for each spring and autumn period, dating back from the spring of 1983 up until the spring of 1986. The responses where categorised into the following seven categories: ‘Education’ (upper secondary school, university, or other education); ‘Labour market measures’ (i.e. government/local programmes such as youth placement or youth activities); ‘Full-time employment’; ‘Part-time/precarious employment’ (incl. causal work); ‘Unemployment’; ‘Outside labour market’ (sick leave, parental leave, living abroad, or prison); and ‘Other’ (incl. military service). It was possible to select two alternatives, which rendered it necessary to construct two parts per period (each corresponding to three months). This resulted in a total of 14 states for the part of the sequence that concerned T2.

For T3, the respondents reported their activities for each spring and fall period from the fall of 1986 up until the fall of 1995. It was only possible to select one alternative from the following options: ‘Education’ (university or other education); ‘Labour market measures’; ‘Full-time employment’; ‘Part-time/precarious employment’ (incl. casual work); ‘Unemployment’; ‘Outside labour market’ (sick leave or parental leave); and ‘Other’. A total of 19 states were added to the part of the sequence that concerned T3.

With regard to T4, activities were reported across 24 periods: from the spring of 1996 up until the fall of 2007. The response options were: ‘Labour market measures’; ‘Full-time employment’ (permanent position or business owner); ‘Part-time/precarious employment’ (temporary employments, e.g. project positions, substitute, probationary, on call, or seasonal); ‘Unemployment’; ‘Outside labour market’; and ‘Other’ (incl. education or travel). Thus, no separate category for ‘Education’ was available in the original questionnaire.

There were 42 individuals who had missing information for an entire part of the sequence (i.e. T2, T3, and/or T4). These were excluded from the analysis. The number of individuals with missing states ranged from 7 to 13 across T2, from 29 to 62 across T3, and from 12 to 24 across T4. For these gaps, states were imputed according to the following principles: a) If the gap consisted only of one missing state, the state from the spring or fall of the same year was imputed, b) If the gap consisted of more than one missing state, and the state on each side of the gap was the same, that state was imputed for the entire gap, and c) If the gap consisted of more than one missing state, and the state on each side of the gap was different, the state to the left of the gap was imputed for the left-hand part, whereas the state to the right of the gap was imputed for the right-hand part.

**Fig. 1:**
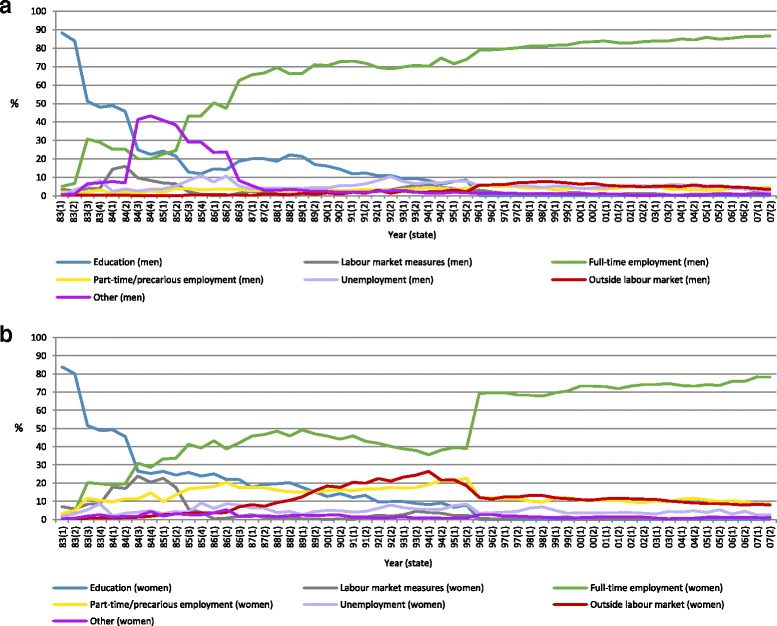
**a**. The distribution of the seven categories of labour market attachment among men (*n* = 492), 1983–2007 (ages 18–42). **b**. The distribution of the seven categories of labour market attachment among women (*n* = 463), 1983–2007 (ages 18–42)

The distribution of the seven categories of education and LMA for each of the 57 states are presented in Fig. 1a (men) and b (women). Figure 1a shows, first of all, that the proportion of men in education decreased continuously over time, apart from a slight bump in the late 80s. The level of unemployment was overall low, apart from the mid-80s, where the prevalence increased to around 15%. Full-time employment increases over time, from approximately 5% at age 18 to almost 80% at age 42. Except for a temporary decrease in the years around 1984 (most likely due to military conscription and unemployment right after graduation from upper secondary school), this increase is quite steep up until the end of the 80s (when the cohort was in their late 20s). The level of part-time/precarious employment was low across the whole measurement period. Rates of unemployment were rather stable around 6%, whereas the proportion of men outside the labour market increased slightly in the early 90s and then remained at stable levels around 6–8%. A high rate of ‘Other’ can be seen in 1984–1986, of which most is likely to reflect military conscription.

Similar to men, the rates of education among women decreased drastically from the beginning of the measurement period and onwards (Fig. 1b). The prevalence of labour market measures was generally low, except for increased levels in years around 1984 (i.e. the period after this cohort would have graduated from upper secondary school). Rates of full-time employment increased across the entire period, except for the first half of the 90s where a significant drop is seen. Part-time/precarious employment was more common among women compared to among men: the prevalence was between 10 and 20% up until the mid-90s, and then becomes stabilised at a level around 10%. The prevalence of unemployment fluctuated slightly across time with average levels around 5%. As previously mentioned, the prevalence of women being outside the labour market increased up until the mid-90s, after which the prevalence remained at a slightly lower level (around 10%). The proportion of women in the category labelled ‘Other’ was low throughout the period.

#### Parental socioeconomic position (age 16)

Parental socioeconomic position was indicated through two types of information: occupational class and employment status. Regarding occupational class, information was reported by participants about whether their father and mother, respectively, were non-manual or manual workers according to the Swedish socioeconomic index (SEI) [61]. Participants were categorised into ‘Low parental occupational class’ if both their mother and father had a manual occupation. Employment status was also based on information from the father and mother respectively, concerning whether they were employed or not. Participants were coded into a category of ‘Parental unemployment’ if they had reported unemployment for at least one parent.

#### Depressive symptoms (age 16)

A scale of depressive symptoms was constructed based on the following six symptoms, experienced over the past 12 months: ‘Poor appetite’, ‘General tiredness’, ‘Concentration difficulties’, ‘Sleeping problems’, ‘Felt down and sad’, and ‘Dejected about future’. These symptoms correspond with the majority of criteria included in the DSM 5 (Diagnostic and Statistical Manual of Mental Disorders, developed by the American Psychological Association). Importantly, however, the scale was not developed as a screening or diagnostic tool [62]. For the purpose of harmonisation, the response options were recoded so that they ranged between 0 and 2 for each item. With regard to ‘Poor appetite’ and ‘General tiredness’, response options were coded into “No” (0), “Yes, mild” (1), and “Yes, severe” (2). The response alternatives for ‘Sleeping problems’, ‘Concentration difficulties’, and ‘Felt down and sad’ were coded as “Never” (0), “On and off” (1), and “Rather often/All the time” (2). For ‘Dejected about the future’, response options were coded as “Never” (0), “Seldom” (1), and “Yes, rather often/Very often” (2). The scale was subsequently computed as the mean of these six items with higher values indicating a higher level of depressive symptoms. This measure has been evaluated and found to present acceptable psychometric properties [62]. In this study, the mean value and standard deviation at age 16 were .48 and .30 for men, and .42 and .28 for women.

### Statistical analysis

As previously mentioned, sequences were only calculated for those who had responded to the question about LMA in all three waves (T2-T4). Therefore, 42 individuals were excluded from the original sample of 1,001 individuals. An additional four individuals had missing information about parental socioeconomic position and depressive symptoms at T1, and were also excluded. The final study sample thus consisted of 955 individuals (males: *n* = 492; females: *n* = 463). Based on labour market information for these individuals, a total of 933 unique sequences were derived, of which the five most prevalent ones are described in Table 1.

**Table 1:** The five most prevalent sequences

Sequences	Percentage
111111111177773333333333333333333333333333333333333333333	0.8
113333777733333333333333333333333333333333333333333333333	0.5
111111111133333333333333333333333333333333333333333333333	0.4
111111777733333333333333333333333333333333333333333333333	0.4
111111111177771111111113333333333333333333333333333333333	0.3

1 = Education; 2 = Labour market measures; 3 = Full-time employment; 4 = Part-time/precarious employment; 5 = Unemployment; 6 = Outside labour market; 7 = Other

The analysis was carried out separately for men and women in three subsequent steps by means of Stata version 13. As a first step, we mapped the individual trajectories of education and LMA through sequence analysis, using the dynamic Hamming algorithm. Cluster analysis was subsequently applied in order to identify typical trajectories. Typical trajectories represent groups of trajectories as suggested in previous research [18]. Next, multinomial regression analysis was used to examine how parental socioeconomic position and depressive symptoms at age 16 was related to the risk of ending up in the various types of trajectory. These steps are described in more detail below.

We used the SADI module in Stata to conduct the sequence analysis. Sequence analysis is based on an algorithmic approach that calculates a matrix of dissimilarities (or distances) between pairs of sequences. The most widely used algorithm is the optional matching (OM) algorithm. It operates in three steps – insertion, deletion, and substitution – as a way of expressing distance, i.e. the minimal amount of effort it takes to change two sequences to become identical [18]. A penalty or ‘cost’ is assigned to each of these steps by the researcher. It is difficult to make theoretically grounded determinations of these costs and, because of that, OM has been criticised [63]. The current study used the dynamic Hamming algorithm [64]. This algorithm has been proposed as an alternative to OM since it does not require any calculations costs related to insertions or deletions (because of that, however, it can only handle sequences of equal length) whereas substation costs are based solely on the data [19].

The sequences were grouped into typical trajectories using a hierarchical cluster analysis of the dissimilarities matrix. Since conventional test statistics are unavailable for sequence data, the determination of the number of clusters must rely on other arguments. The number in the present study was determined by the observation of theoretically meaningful clusters, saturation (i.e. when the addition of a new cluster was only a version of an already existing cluster), and a sufficient number of cases in each cluster [21]. Based on these principles, it was decided to proceed with the four-cluster solution for men and the five-cluster solution for women. All participants were assigned to a cluster (typical trajectory).

Due to the multi-categorical nature of the outcome, multinomial regression analysis was used to examine the associations between parental socioeconomic position, depressive symptoms, and the type of trajectories of education and LMA. The typical trajectories showing the highest level of education together with stable full-time employment were used as reference categories because those individuals were, based on existing evidence [6], assumed to be best off socioeconomically and health wise. The analysis was modelled in two steps: the first step investigated the crude (i.e. unadjusted) associations from low parental occupational class, parental unemployment, and depressive symptoms to typical trajectories. The second step included a series of mutually adjusted models of which the first examined low parental occupational class and depressive symptoms at the same time (Part I), while the next encompassed both parental unemployment and depressive symptoms (Part II). The subsequent model simultaneously included all three independent variables in relation to typical trajectories (Part III).

## Results

### Education and labour market attachment trajectories among men

The typical education and LMA trajectories are illustrated by means of chronograms (graphs of the time-dependent distribution of education and LMA; one per type of trajectory). Beginning with men (Fig. 1a), the first typical trajectory ‘Long education into stable employment’ contained 29.1% (*n* = 143) of the individuals. It is characterised by a long duration of education (with a hiatus for military service, as reflected by the purple segment) and the employment levels seem to be stable from the individuals’ mid-20s (from the beginning of the 1990s) and onwards.

The typical trajectory ‘Medium education into stable employment’ consists of 22.2% (*n* = 109) of the men. Men following this typical trajectory were in the educational system for a shorter period of time compared to the previous typical trajectory, followed by stable employment.

‘Short education into stable employment’ characterised the third and largest typical trajectory (31.5%, *n* = 155). With the exception of military service and some experience of labour market measures in late adolescence, these men were in stable employment through the period.

The fourth and smallest typical trajectory is labelled ‘Continuously unstable situation’ (17.3%, *n* = 85). Throughout the whole period, it was common for men to experience comparably high levels of labour market measures, part-time/precarious employment, unemployment, and being outside the labour market. With regard to education, these men seem to have spent in education than those in the previous typical trajectory.

### Education and labour market attachment trajectories among women

**Fig. 2:**
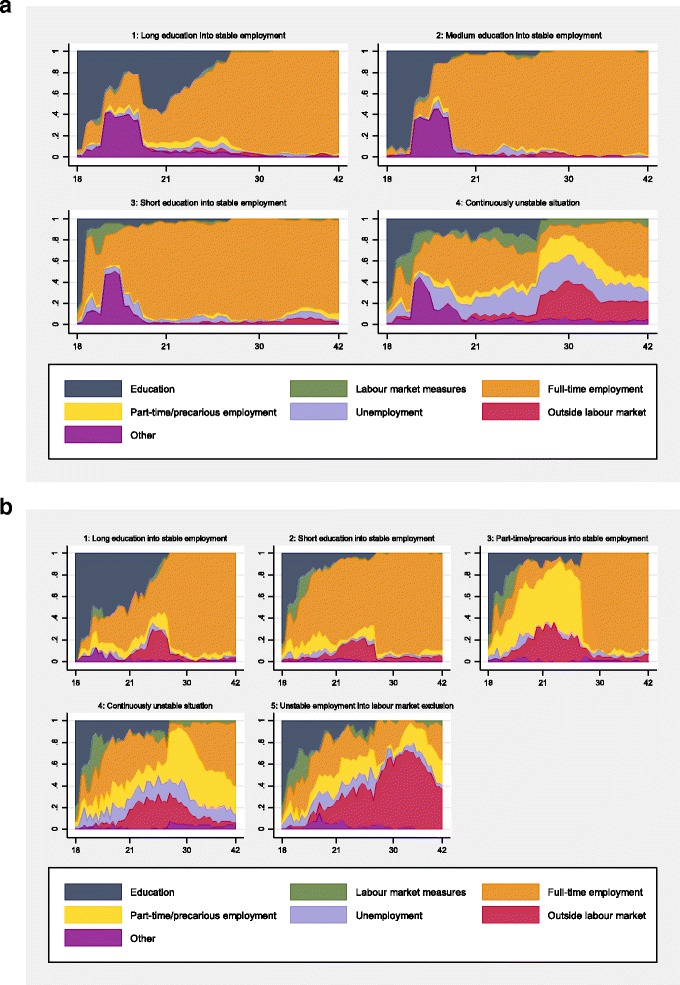
**a**. Typical trajectories of education and labour market attachment among men (*n* = 492), across ages 18–42 (1983–2007). Note: Trajectory 1: 29.1% (*n* = 143); Trajectory 2: 22.2% (*n* = 109); Trajectory 3: 31.5% (*n* = 155); Trajectory 4: 17.3% (*n* = 85). Y-axis shows the proportion of individuals, x-axis shows age. **b**. Typical trajectories of education and labour market attachment among women (*n* = 463), across ages 18–42 (1983–2007). Note: Trajectory 1: 18.1% (*n* = 84); Trajectory 2: 37.4% (*n* = 173); Trajectory 3: 15.6% (*n* = 72); Trajectory 4: 17.3% (*n* = 80); Trajectory 5: 11.7% (*n* = 54). Y-axis shows the proportion of individuals, x-axis shows age

Figure 2b shows the chronograms for the typical trajectories of women’s education and LMA. The first typical trajectory, ‘Long education into stable employment’ contained 18.1% (*n* = 84) of the women. There was some presence of part-time/precarious employment throughout the period and, moreover, there was a temporary increase of being outside the labour market in these women’s mid-20s (beginning of the 1990s). This could potentially reflect the major economic recession in Sweden during this time. Following this, employment was stable.

The second typical trajectory – ‘Short education into stable employment’ – was the largest and contained 37.4% (*n* = 173) of the women. It was similar to the previous typical trajectory but was characterised by a shorter period of education.

About 15.6% (*n* = 72) of the women followed the third typical trajectory: ‘Part-time/precarious into stable employment’. It was characterised by a mix of different states, including education, across late adolescence and the beginning of early adulthood, particularly in terms of part-time/precarious employment. After this, the vast majority experienced full-time employment.

The fourth typical trajectory was characterised by a ‘Continuously unstable situation’, with comparably high levels of part-time/precarious employment, unemployment, and being outside the labour market throughout most of the period. The proportion of time spent in education was similar to that of the second typical trajectory. From their 30s (mid-1990s) and onwards, nevertheless, a majority were in employment – either full-time or part-time.

The smallest typical trajectory ‘Unstable employment into labour market exclusion’ consisted of 11.7% (*n* = 54) of the women. Similar to the previous typical trajectory, it was characterised by several, presumably short, periods of study, comparably high levels of part-time/precarious employment, unemployment, and being outside the labour market. However, when these women reached their late 20s (end of the 1990s), being outside the labour market seemed to be the predominant situation.

### Parental socioeconomic position, depressive symptoms, and typical trajectories among men

In Table 2, the percentage distribution of low parental occupational class and parental unemployment as well as mean values of depressive symptoms in adolescence are presented for each of the typical trajectories of education and LMA among men.

**Table 2:** Percentage distribution of low parental occupational class, parental unemployment, and mean values of depressive symptoms across typical trajectories. Results for men (*n* = 492)

	Trajectory 1	Trajectory 2	Trajectory 3	Trajectory 4
Low parental occupational class (%)	23.1	28.4	53.6	54.1
Parental unemployment (%)	12.6	22.9	23.2	25.9
Depressive symptoms (mean)	0.42	0.36	0.42	0.50

Trajectory 1: Long education into stable employment (29.1%, *n* = 143); Trajectory 2: Medium education into stable employment (22.2%, *n* = 109); Trajectory 3: Short education into stable employment (31.5%, *n* = 155); Trajectory 4: Continuously unstable situation (17.3%, *n* = 85)

Table 3 shows how parental occupational class, parental employment status, and depressive symptoms at age 16 were associated with the four typical trajectories of education and LMA among men. The upper section of the table concerns the unadjusted associations whereas the lower section includes a series of mutually adjusted models (Parts I–III).

**Table 3:** Parental occupational class and employment status (age 16), depressive symptoms (age 16), and typical trajectories of education and labour market attachment (ages 18–42) among men (*n* = 492)

	Trajectory 1 (ref.)	Trajectory 2	Trajectory 3	Trajectory 4
	RRR (95% CI)	RRR (95% CI)	RRR (95% CI)	RRR (95% CI)
Unadjusted				
Low parental occupational class	1.00	1.32 (0.75, 2.34)	**3.84** (2.33, 6.34)	**3.93** (2.21, 7.00)
Parental unemployment	1.00	**2.07** (1.06, 4.02)	**2.10** (1.13, 3.90)	**2.43** (1.21, 4.85)
Depressive symptoms	1.00	0.45 (0.18, 1.10)	0.95 (0.43, 2.07)	**2.48** (1.02, 6.02)
Mutually adjusted, Part I				
Low parental occupational class	1.00	1.36 (0.76, 2.40)	**3.86** (2.34, 6.38)	**3.82** (2.14, 6.82)
Depressive symptoms	1.00	0.44 (0.18, 1.08)	0.85 (0.38, 1.90)	2.20 (0.89, 5.46)
Mutually adjusted, Part II				
Parental unemployment	1.00	**2.40** (1.21, 4.76)	**2.18** (1.16, 4.10)	**2.17** (1.07, 4.41)
Depressive symptoms	1.00	**0.36** (0.14, 0.90)	0.78 (0.35, 1.73)	2.03 (0.82, 5.05)
Mutually adjusted, Part III				
Low parental occupational class	1.00	1.30 (0.73, 2.32)	**3.74** (2.26, 6.19)	**3.69** (2.06, 6.61)
Parental unemployment	1.00	**2.33** (1.18, 4.61)	**1.97** (1.03, 3.76)	1.94 (0.94, 4.01)
Depressive symptoms	1.00	**0.36** (0.14, 0.90)	0.72 (0.32, 1.66)	1.89 (0.74, 4.79)

Relative risk ratios (RRRs) from multinomial regression analysis, with 95% confidence intervals (CI) within brackets. Statistically significant estimates (*p* < 0.05) in bold

Trajectory 1: Long education into stable employment (29.1%, *n* = 143); Trajectory 2: Medium education into stable employment (22.2%, *n* = 109); Trajectory 3: Short education into stable employment (31.5%, *n* = 155); Trajectory 4: Continuously unstable situation (17.3%, *n* = 85)

With regard to the unadjusted association between parental occupational class and LMA, the results show that individuals, who grew up in families where the parents had a low occupational class, had a higher risk of ending up in other typical trajectories than that of long education and stable employment. For example, the individuals in this group have a relative risk ratio of 3.84 (95% CI: 2.33, 6.34) of ending up in the typical trajectory characterised by ‘Short education into stable employment’ (Typical trajectory 3). The risk is almost four-fold (RRR: 3.93; 95% CI: 2.21, 7.00) also for ending up in typical trajectory 4, which denotes a ‘Continuously unstable situation’. Moreover, while there is an increased risk of ending up in typical trajectory 2 (RRR: 1.32), this estimate is not statistically significant.

For parental employment status, individuals who had at least one unemployed parent at age 16 were more than twice as likely to ending up in typical trajectories 2–4 compared to typical trajectory 1. The estimates were rather similar (RRRs ranging from 2.07 to 2.43), suggesting that the biggest gap is between the typical trajectory characterised by high education and stable employment (typical trajectory 1) and the remaining typical trajectories.

With regard to the unadjusted association between depressive symptoms and the typical trajectories of education and LMA, a higher level of depressive symptoms was linked to lower risks of ending up in typical trajectories 2 and 3. However, these estimates were not statistically significant. An increased risk was found for typical trajectory 4: men with higher levels of depressive symptoms had a relative risk ratio of 2.48 (95% CI: 1.02, 6.02) of ending up in the typical trajectory characterised by a ‘Continuously unstable situation’.

In the mutually adjusted model, Part I, the increased risks for men whose parents had a low occupational class of ending up in typical trajectories 3 and 4 remained statistically significant. The estimate for depressive symptoms regarding typical trajectory 4 was however reduced to a statistically non-significant level. Regarding Part II, men who had at least one unemployed parent still had higher risks of ending up in typical trajectories 2–4, although the RRRs changed slightly (upwards for typical trajectory 2 and 3; downwards for typical trajectory 4). Concerning depressive symptoms, the estimate for typical trajectory 4 was no longer statistically significant. Interestingly enough, however, the negative risk of ending up in typical trajectory 2 was now at a significant level (RRR: 0.36; 95% CI: 0.14, 0.90), indicating that men with higher levels of depressive symptoms are less likely to end up in the typical trajectory characterised by medium levels rather than high levels of education (in combination with subsequent stable employment). In Part III, which includes all three independent variables simultaneously, the overall results from Parts I and II are largely reproduced.

### Parental socioeconomic position, depressive symptoms, and typical trajectories among women

Table 4 displays the percentage distribution of low parental occupational class and parental unemployment as well as mean values of depressive symptoms in adolescence for each typical trajectory of education and LMA among women.

**Table 4:** Percentage distribution of low parental occupational class, parental unemployment, and mean values of depressive symptoms across typical trajectories. Results for women (*n* = 463)

	Trajectory 1	Trajectory 2	Trajectory 3	Trajectory 4	Trajectory 5
Low parental occupational class (%)	15.5	34.7	41.7	45.0	51.9
Parental unemployment (%)	15.5	13.9	19.4	22.5	27.8
Depressive symptoms (mean)	0.51	0.56	0.54	0.57	0.59

Trajectory 1: Long education into stable employment (18.1%, *n* = 84); Trajectory 2: Short education into stable employment (37.4%, *n* = 173); Trajectory 3: Part-time/precarious into stable employment (15.6%, *n* = 72); Trajectory 4: Continuously unstable situation (17.3%, *n* = 80); Trajectory 5: Unstable employment into labour market exclusion (11.7%, *n* = 54)

Table 5 demonstrates the associations between parental occupational class, parental employment status, depressive symptoms, and typical trajectories of education and LMA among women. Women whose parents had a low occupational class showed increased risks of ending up in typical trajectories 2–5 compared to typical trajectory 1. In comparison to the results for men, the relative risk ratios were overall much higher. For example, the estimate for typical trajectory 2 was almost three-fold (RRR: 2.90; 95% CI: 1.49, 5.66) and the estimate for typical trajectory 3 was nearly four-fold (RRR: 3.90; 95% CI: 1.83, 8.30). For typical trajectory 4, the relative risk ratio was even higher (RRR: 4.47; 95% CI: 2.14, 9.34) – only exceeded by the one for Typical trajectory 5 (RRR: 5.88; 95% CI: 2.65, 13.0). Unlike for men, there were no statistically significant estimates with regard to parental employment status and the typical trajectories of education and LMA, although the tendency for women with unemployed parents was to have higher risks of ending up in typical trajectories 3–5. The relative risk ratios for the association between depressive symptoms and the typical trajectories of education and LMA varied between 1.38 and 2.65 for typical trajectories 2–5. However, none of the estimates reached a statistically significant level.

**Table 5:** Parental occupational class and employment status (age 16), depressive symptoms (age 16), and typical trajectories of education and labour market attachment (ages 18–42) among women (*n* = 463)

	Trajectory 1 (ref.)	Trajectory 2	Trajectory 3	Trajectory 4	Trajectory 5
	RRR (95% CI)	RRR (95% CI)	RRR (95% CI)	RRR (95% CI)	RRR (95% CI)
Unadjusted					
Low parental occupational class	1.00	**2.90** (1.49, 5.66)	**3.90** (1.83, 8.30)	**4.47** (2.14, 9.34)	**5.88** (2.65, 13.0)
Parental unemployment	1.00	0.88 (0.42, 1.83)	1.32 (0.57, 3.03)	1.59 (0.72, 3.50)	2.10 (0.91, 4.86)
Depressive symptoms	1.00	1.82 (0.71, 4.64)	1.38 (0.45, 4.28)	2.12 (0.71, 6.34)	2.65 (0.79, 8.94)
Mutually adjusted, Part I					
Low parental occupational class	1.00	**2.86** (1.46, 5.59)	**3.87** (1.82, 8.25)	**4.40** (2.10, 9.20)	**5.77** (2.60, 12.8)
Depressive symptoms	1.00	1.69 (0.66, 4.28)	1.26 (0.40, 3.91)	1.92 (0.64, 5.80)	2.39 (0.69, 8.24)
Mutually adjusted, Part II					
Parental unemployment	1.00	0.83 (0.40, 1.74)	1.28 (0.56, 2.97)	1.49 (0.67, 3.30)	1.94 (0.83, 4.53)
Depressive symptoms	1.00	1.86 (0.73, 4.78)	1.32 (0.42, 4.13)	1.98 (0.65, 5.97)	2.35 (0.68, 8.08)
Mutually adjusted, Part III					
Low parental occupational class	1.00	**2.98** (1.52, 5.87)	**3.87** (1.80, 8.29)	**4.31** (2.04, 9.09)	**5.47** (2.44, 12.3)
Parental unemployment	1.00	0.70 (0.33, 1.48)	1.02 (0.43, 2.41)	1.16 (0.51, 2.64)	1.44 (0.60, 3.46)
Depressive symptoms	1.00	1.78 (0.70, 4.54)	1.25 (0.40, 3.92)	1.87 (0.61, 5.69)	2.21 (0.63, 7.77)

Relative risk ratios (RRRs) from multinomial regression analysis, with 95% confidence intervals (CI) within brackets. Statistically significant estimates (*p* < 0.05) in bold

Trajectory 1: Long education into stable employment (18.1%, *n* = 84); Trajectory 2: Short education into stable employment (37.4%, *n* = 173); Trajectory 3: Part-time/precarious into stable employment (15.6%, *n* = 72); Trajectory 4: Continuously unstable situation (17.3%, *n* = 80); Trajectory 5: Unstable employment into labour market exclusion (11.7%, *n* = 54)

In the mutually adjusted models (Parts I–III), the estimates for parental occupational class are slightly reduced but remain statistically significant. The results for depressive symptoms and parental unemployment changed only marginally and all estimates remained non-significant.

## Discussion

The present study, based on a Swedish cohort born in 1965 which was followed from age 16 to 42 years, aimed to identify typical life-course trajectories of education and LMA in 18–42 year old men and women and examine to what extent parental socioeconomic position and level of youth depressive symptoms could predict which type of trajectory the individuals would follow. The novelty of the study is mainly represented by the long follow-up period with low attrition rates, as well as the complexity of data in terms of the number of states underlying the sequences. To our knowledge, no other study has mapped the education and LMA trajectories from late adolescence into mid-life with such rich data. Exploring how depressive symptoms in youth are related to which trajectory people follow is also relatively unique within the field.

We identified four typical trajectories for men and five for women. In men, compared to those in the trajectory of long education and stable employment, those following the short education or the continuously unstable trajectories were more likely to have had parents with a low occupational class position or parents who were unemployed. Overall, the level of youth depressive symptoms showed non-significant differences regarding membership across the trajectories. Adjusting for depressive symptoms did not significantly alter the associations between parental socioeconomic position and the life-course trajectories of education and work.

The findings show support for an inter-generational social reproduction also in women, although associations were only found when parental socioeconomic status was operationalised as occupational class (as opposed to unemployment). Similar to men, depressive symptoms did not differ significantly across the trajectories, nor did the adjustment for depressive symptoms explain the associations between parental occupational class and the trajectories of education and labour market attachment. Hence, we fail to conclude that intergenerational social reproduction is linked to depressive symptoms in adolescence among women.

### Trajectories

The following discussion will mainly focus on inter-generational social reproduction and mental health selection. However, we will first discuss the trajectories that were identified. The characteristics of education and LMA trajectories, in the current as well as other studies, depend highly on socio-historical circumstances [21] as well as the period under investigation. While the current study treats the issue of time primarily in regard to age, it should be emphasised that age effects are very difficult to separate from cohort and period effects. For example, over the past decades macro-social and macro-economic conditions have undergone major changes, with implications for making life courses less conventional [29]. The individuals examined in the present study, were teenagers and young adults in a time of a relatively stable labour market, just before the economic crisis in the early 1990s with sharp increase of youth unemployment [60]. Compared to most other West European countries Sweden was less severely hit by mass unemployment in the 1970s and 1980s [55].

Despite the specific characteristics of the socio-historical context in which the lives of the Northern Swedish Cohort members are imbedded, our results are similar to other studies. For example, according to findings on women from the British Cohort Study, the participants followed trajectories of: Part-time employment; Unemployment; High level education; and Detachment from the labour market [8]. Similarly, Widmer et al. [28] identified occupational trajectories characterised by: Full-time employment; Mixed occupations; Return to part-time employment after decline in full-time ditto, At home trajectories; and Part-time employment, in Swiss adults between age 20 and 45. A longitudinal Scottish study of young people aged 16 to 23 years (of a cohort born in 1973), found eight transitional trajectories of education and employment: Long higher education; Short higher education; Enhanced education; Direct job; Assisted employment; Unemployment; Domestic; and Other [26]. Another study applying sequence analysis identified the following youth-to-adulthood-transitions: Employment; Higher education; Further education; Joblessness; and Employment after long further education or training [12]. Across all these studies, including ours, stable employment dominates whereas a minority of individuals follow a trajectory which initially is unstable but then becomes more stable with regard to employment. It also seems to be common across studies to identify a group of disadvantaged individuals with regard to short education and positions outside the labour market.

Although the distinct features of the trajectories for men and women are in line with previous findings [28], they justify a further discussion. Men in our cohort tended to have more stable and less diverse trajectories of education and LMA than women. These differences are likely to be due to differences in possibilities for labour market participation and domestic responsibilities, including caring for children. For example, despite the gender equality regime of the Scandinavian countries, the Swedish labour market is remarkably gender segregated with men dominating the private sector and manufacturing industry, and women the public sector [58, 59]. Public sector jobs are also more likely to be part-time and with a significantly lower pay compared to the male dominated sectors. Furthermore, Sweden has a generous parental leave cash benefit system for care of children as well as work time reduction for parents [65]. Even though the system is not targeting mothers only, women use the majority of the benefits, with implication of weakening of the labour market attachment [65]. Women, especially those working in the public sector, are also more likely than men to be on long-term sick leave [66]. The analyses rendered trajectories of instability (or disadvantage) in both men and women, but different patterns were shown regarding precariousness. For example, in men it seems like those hit by unemployment were concentrated in one typical trajectory whereas unemployment was more spread out across trajectories in women. The activity state ‘outside the labour market’ was also more dominant among women than men, which, as noted above, most likely due to parental leave and sick leave. These findings are similar to those of Sweeting et al. [6] based on a Scottish cohort of participants born in 1972 where the main reason for non-employment among men was unemployment and among women caring for home or family. The proportion of full-time employment in women is lower than among men, which most likely is due to women working part time and taking more responsibility for unpaid labour in the home [65, 67, 68]. In 1994/95, at 30 years of age, approximately 17% of women and 1.5% of men in this cohort reported that they were on parental leave (data not presented).

There is a previous study of the Northern Swedish Cohort (8) which assessed LMA from age 30 to 42 by an ordinal scale variable (permanent employment, temporary employment, unemployment, inactivity) and searched the trajectories by latent class growth analysis [69]. In line with the pattern of the present study, the trajectory of high level LMA was predominant in both genders, but women tended to assume more commonly the trajectories of strengthening or medium level LMA. Thus, as regards to LMA only, these different methodological approaches seem to yield largely similar results.

### Prediction of trajectories

We found support for the hypothesis of inter-generational social reproduction, which is also in line with previous research using sequence analysis to identify education and work trajectories [8–10, 12]. In other words, if the parents of a teenager have low occupational status and/or are unemployed, the teenager is more likely to follow trajectories of short education and employment instability compared to peers whose parents are better off. It should be noted that the strength of the associations seemed to vary between men and women. However, the gender-specific results cannot be directly compared since the typical trajectories were not the same.

Another and perhaps even more interesting finding was the heterogeneity within the categories of men and women with regard to which indicator of parental socioeconomic position that was considered. Men who had experienced parental unemployment showed a two-fold risk of ending up in the all other trajectories than “Long education into stable employment”. The relative differences across the trajectories regarding low parental occupational class were relatively larger – almost four-fold – but only present for the two least advantaged trajectories. Also among women, both indicators of parental socioeconomic position seemed to be associated with an increased risk of ending up in the less advantaged education and work trajectories. However, while these results were clear and statistically significant for parental occupational class, the differences were not statistically significant for parental employment status. These disparate findings – particularly evident for women – could be a consequence of the lack of statistical power, but could also be interpreted based on the differential meaning of occupational class and employment status of the parents for their offspring. Regarding parental occupational class, it is likely to reflect the general level of resources in the household and may be seen as a relatively stable condition. Employment status, on the other hand, will for most reflect a more temporary situation which mainly affects the availability of economic resources. Since the educational system is free of charge for all Swedish citizens, the family’s economic resources may not be as important for the youth’s future education and work trajectories as compared to the social and educational capital attached to the occupational class of the parents.

Depressive symptoms in youth showed no, or very limited, links with subsequent education and work trajectories also when taking parental socioeconomic position into account. The unadjusted model for men showed that those who reported elevated levels of depressive symptoms in youth were more likely to follow the most disadvantaged typical trajectory compared to those with the longest education and stable employment. This relates to existing research showing that men with poorer health are at risk of downward mobility [41] but contrasts the findings by Birkeland et al. [11] who found support for mental-health related social selection for girls only. However, our results were unstable and the overall conclusion from the analyses is, in accordance with some other studies [32, 42], that there is poor evidence of effects of youth depressive symptoms on adult typical trajectories of education and LMA. At the same time, and rather surprisingly, when adjusting for parental unemployment, depressive symptoms in adolescence showed *reduced* likelihood of following the trajectory of medium education into stable employment compared to the reference category in men. Are young men who intend to continue their studies more likely to report depressive symptoms than their less ‘study inclined’ male peers? It is possible that this is a reflection of being harassed for not adhering to hegemonic norms of masculinity in which being ‘studious’ does not fit [70]. It is also possible that the longer education in men who had unemployed parents is a result of poorer mental health, for example that they had difficulties completing their degree, lacked motivation, or were enrolled in courses without really wanting to. Further speculations may be that these young men wished to work but were discriminated against at the labour market because of poor mental health and that they therefore chose to study.

Our results regarding the lack of association between level of youth depressive symptoms and trajectory membership can be understood from several perspectives. First, it might reflect a buffer effect of the educational system in Sweden. Although many young people experience depressive symptoms, the most common pathway for young people in the early 1980s was to continue to upper secondary school/senior high school after nine years of compulsory schooling (80% of 16-year olds) [71]. Hence, there was a low risk of school drop-out which has been found to increase the risk of labour market exclusion and long-term welfare benefit dependency [72]. In addition, research suggests an ‘equalising effect’ of school regarding the social gradient in health [73]. According to this hypothesis, socioeconomic differences in health are greater before and after compulsory education, suggesting that schooling and the school environment may decrease the socioeconomic gap in health during this phase of life. If young people have the chance to continue in school despite elevated levels of depressive symptoms, it is possible that the selection effect is reduced.

Second, the findings might also reflect the buffering effect of the Swedish welfare state, providing a foundation of social and economic protection, for example via free health care and active labour market policies. For example, the school health care was well developed in Sweden at this time and all upper secondary schools/senior high schools had school nurses and counsellors employed to which young people could turn if they needed support for mental health troubles. The low level of youth unemployment at the time, together with the previously mentioned national policies regarding labour market programs, might also have contributed to a sense of security and predictability among young people and less discrimination of individuals with mental health problems than would have been the case in a more competitive insecure situation.

Third, it also possible that the depressive symptoms experienced at age 16 years (for both boys and girls) were not severe, long-term, or frequent enough to show lasting effects on their educational and employment-related choices later on in life. The symptoms assessed here represent relatively minor problems as opposed to, for example, clinically diagnosed major depression, which has been found to have long-term effects on people’s life chances [14, 15]. Perhaps the level of depressive symptoms in youth has more immediate consequences or is related to other outcomes in their future lives than education and LMA. For example, according to a study based on the same cohort, men with mental health problems in youth were less likely to become fathers [74]. It is also possible that the health selection effects are more prominent later in life, as identified in previous research [34, 40, 47, 49].

Nevertheless, despite the lack of significant associations between depressive symptoms and the typical education and LMA trajectories identified, we would like to point out that the estimates suggest a positive association, that is, the higher level of depressive symptoms in youth, the greater likelihood of following a less fortunate typical trajectory than the one dominated by long education and stable employment. In the case of men, the unadjusted analyses showed that those following the typical trajectory of disadvantage (the continuously unstable trajectory) were more likely to report elevated levels of depressive symptoms than their peers in the long education and stable employment trajectory. This indicates health selection, reflecting what life-course epidemiology would call a ‘chain of risk’ [75]. However, the adjusted analyses suggest that this association depends on parental socioeconomic position.

To the extent it indicates no discrimination of individuals with mental health problems, it is positive that youth level of depressive symptoms shows a weak association with adult status regarding education and LMA. Our findings suggest that typical trajectory membership is influenced by other factors than depressiveness in youth. This suggests that future research should focus on the importance of indirect selection processes as concluded by Foreskov et al’s analyses of the British household panel [76]. A next step is to analyse mental health consequences of typical trajectory membership while taking both inter-generational and intra-generational social reproduction into account.

### Strengths and limitations

The main strengths of the current study are the long-term cohort material covering 26 years, the low attrition, and the large number of states to model in the sequence analyses. The low attrition implies that those who are most likely to drop out, for example individuals with poor health [77, 78], are still represented. Nevertheless, some limitations are worth noticing. For example, there is a risk of recall bias because data on activity was reported retrospectively, especially for the nine and twelve year periods between age 21 and 30 as well as 30 and 42. For the calculation of the individual sequences, gaps were imputed based on the states before and after the gap. Although commonly used, this strategy could have overestimated the stability of the trajectories. All data was self-reported which implies a risk of positive and negative affectivity, i.e. that individuals in a negative mood state may recall and report ‘worse’ activities (for example unemployment or sick-leave) than individuals in a more positive mood states [79, 80].

With regard to the measures used, the variable of seven categories that reflects education and labour market attachment is relatively crude and restricts the variation in individual trajectories. It is also a limitation that the response categories differed across waves, for example that the option ‘education’ was not available for the period between age 30 and 42. Moreover, information on parental income and level of education would have facilitated a more complex analysis of intergenerational social reproduction. Unfortunately, such data were not available. Finally, depressive symptoms is only one aspect of mental health and although the measure used in the current study shows good psychometric properties and corresponds well with symptoms included in the DSM 5 manual, there is always a risk that the findings are strongly dependent upon the measure used. It is possible that the results would have been different had we used other indicators of (mental) health. Thus, our zero finding about mental health selection should be generalised with caution.

## Conclusions

This study adds to the literature base in respect of mapping complex trajectories of education and labour market attachment in a 26-year prospective study. It further contributes by confirming the hypothesis of intergenerational reproduction of social position, particularly when measured in terms of parental occupational class. Interestingly, no evidence of health-related social selection was found, as the level of depressive symptoms at age 16 had little or no crude association with future education and LMA trajectories. While this could be a consequence of the specific framework of the current study, it may also suggest that depressive symptoms in youth are not a root cause for the more complex processes through which how social and labour market positions develop across life. The lack of health-related social selection could be a consequence of the Scandinavian welfare regime, including the school system and labour market policies, as well as macro-economic circumstances. We conclude that deeper understandings of the antecedents of life-course trajectories of education and work require complex analyses of the social context, including how life circumstances differ between men and women. Interventions to improve the life chances and mental health of young people should acknowledge these conclusions, although more research is warranted.

## Abbreviations

CI: Confidence interval
DSM: Diagnostic and statistical manual of mental disorders
LMA: Labour market attachment
OM: Optimal matching
RRR: Relative risk ratio
SEI: Socioeconomic index
T1: Time 1
T2: Time 2
T3: Time 3
T4: Time 4
